# Association between winter season and desmopressin treatment efficiency in children with monosymptomatic nocturnal enuresis: a pilot study

**DOI:** 10.1590/S1677-5538.IBJU.2021.0236

**Published:** 2022-01-10

**Authors:** Mengkui Sun, Shoulin Li, Xuerui Sun, Zhimei Deng, Yanan Xu

**Affiliations:** 1 Shenzhen Children's Hospital Department of Urology Shenzhen China Shenzhen Children's Hospital, Department of Urology, Shenzhen, China

**Keywords:** Nocturnal Enuresis, Treatment Failure, Child

## Abstract

**Objectives:**

The purpose of our study was to assess the association between the winter season and desmopressin treatment failure in South Chinese children with monosymptomatic nocturnal enuresis (MNE).

**Materials and Methods:**

A retrospective study was conducted to analyze the clinical data of children with monosymptomatic nocturnal enuresis who have visited our urology clinic from January to December 2019. All patients received desmopressin treatment. Final treatment outcomes were categorized as successful (complete response) or failed (absent and partial response). The relationship between winter season and treatment response to desmopressin was evaluated. Additionally, associated risk factors were investigated with both univariate and multivariate regression analysis.

**Results:**

In total, 393 patients diagnosed with MNE were included in the present study. There were no statistically significant differences in pretreatment variables at first visit between patients who visited the clinic in winter and those who did so in other seasons. However, the treatment failure rate of MNE in the winter season was higher than that of other seasons (77.50% vs. 52.74%). Multivariate logistic regression analysis demonstrated that the severity of symptoms and an initial clinic visit in the winter season were significantly related to desmopressin treatment failure in MNE patients.

**Conclusion:**

Winter season and severity of symptoms are two risk factors associated with desmopressin treatment failure in MNE patients.

## INTRODUCTION

Nocturnal enuresis (NE) is defined as intermittent incontinence during sleep that manifests in at least one episode per month for three consecutive months in children older than five years (1). NE is a common developmental disorder, especially in boys, and its prevalence among children aged 7 is 5-10% (2, 3) and among adults is 0.5-2.3% (4, 5).

Enuresis can be classified as monosymptomatic nocturnal enuresis (MNE) or non-monosymptomatic nocturnal enuresis (NMNE) depending on whether accompanying lower urinary tract symptoms are present, with the former type being characterized by the absence of such symptoms. Desmopressin, a synthetic version of the human antidiuretic hormone (ADH), is one of the recommended first-line treatments for MNE (6). In fact, desmopressin reduces nocturnal urine production, matching such production with bladder capacity. After receiving oral desmopressin treatment, nearly one-third of children had no subsequent MNE episodes, one-third obtained partial relief, and one-third had no benefit (7). MNE cure rate can be improved by combination therapy strategies (8).

The winter season was reported to be a risk factor in enuresis treatment failure in Japan (9). The same trend was also observed in Shenzhen, a city that belongs to the subtropical monsoon climate zone in southern China, but the nature of the relationship between the winter season and desmopressin treatment failure remains unclear. Therefore, in our current study, we investigated the difference between children who received desmopressin treatment in the winter and those who received it during other seasons.

## MATERIALS AND METHODS

### Participant selection

We retrospectively analyzed the clinical data of 460 consecutive children diagnosed with MNE who had been referred to urology outpatient clinic of Shenzhen Children's Hospital between January to December 2019. All procedures performed in this study were in accordance with the ethical standards of the Research Ethics Committee of Shenzhen Children's Hospital (IRB number: 2021044). All included children with MNE met the diagnostic criteria listed by the International Children's Continence Society (ICCS): age >5 years; involuntary nocturnal leakage at least once a month; and duration of MNE >3 months (10, 11). Patients were excluded if they presented with one of the following exclusion criteria: organic lesions including neurological or urinary system malformations, diabetes mellitus; concomitant daytime symptoms; and treatment duration spanning two time points (December to February and March to November). Patients with missing information (n=30) and those who had poor compliance (n=37) were excluded from the study. Ultimately, the data of 393 children with MNE were analyzed.

**Figure 1:**
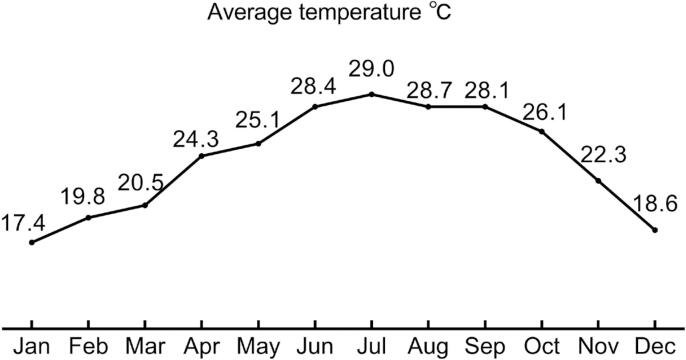
The average temperature (°C) in Shenzhen region in 2019. Data were recorded from the Shenzhen Meteorological Bureau website (http://weather.sz.gov.cn/qixiangfuwu/qihoufuwu/qihouguanceyupinggu/nianduqihougongbao/content/post_6851743.html).

The severity of enuresis was classified based on the number of nights when MNE was observed: mild, less than 2 nights per week; moderate, 3-4 nights per week; and severe, greater than 5 nights per week (12). Shenzhen is located in the southeast coast of China, close to Hong Kong, with a typical subtropical monsoon climate. Therefore, the winter season was considered to span from December to February (Figure-1).

All patients received routine health coaching about avoiding irregular bladder and bowel habits, establishing healthy lifestyle behaviors and limiting fluid intake before bedtime during desmopressin treatment. All children received desmopressin tablets as first-line treatment and were followed up for a minimum of three months. The initial dose of desmopressin was 0.1mg 1h before bedtime. This dose could be raised to a maximum dose of 0.4mg if patients showed no response after two weeks. The treatment response was evaluated after three months’ treatment, and therapy was discontinued if possible. Treatment unresponsiveness, partial responsiveness and complete responsiveness, was defined as a reduction in symptom frequency less than 49%, equal to 50-99%, and equal to 100% dry nights, respectively (1). Treatment failure was defined as: absent or partial response (13) and the patients were divided into a successful or failed outcome group according to their responsiveness.

### Statistical Analysis

The SPSS 25.0 statistical software (SPSS Inc., Chicago, IL, USA) was used for statistical analysis. A chi-square test was employed to analyze the relationship between variables of the two groups, a t test was utilized for computing statistical differences, and the Mann-Whitney U test was used to compare treatment efficacy between the two groups. Statistical analyses were performed using univariable logistic regression followed by multivariable logistic regression to evaluate possible risk factors of desmopressin treatment failure. P values <0.05 were considered statistically significant.

## RESULTS

A total of 393 children diagnosed with MNE were included in our current study. The mean age of the cohort was 7.39 years, and the mean follow-up time was 6.3 months after treatment initiation. A total of 206 children (52.42%) presented with severe symptoms according to frequency of nighttime urination, and 117 subjects (29.77%) had a positive family history of enuresis. Habitual snoring during sleep was reported by 78 subjects (19.85%). Obesity was observed in 45 (11.45%) patients, while 348 (88.55%) children were classified as non-obese according to BMI (Table-1). There was no difference between the two groups in age, sex, severity of symptoms, family history, parent educational level, sleep quality, and weight problems (Table-1).

**Table 1:** Characteristics of cases for the first visiting urology clinic in winter or other seasons.

Variables	winter n = 120	other seasons n = 273	t/χ2/Z	p
Age (years ± SD)	7.53±1.67	7.34±1.42	1.170	0.243
Gender(male/female)	51/69	143/130	3.256	0.071
Mother's age (years ± SD)	34.42±5.34	34.90±5.83	0.764	0.445
Father's age (years ± SD)	38.55 ±5.79	38.09±6.02	0.715	0.475
Severity of symptoms (severe / mild and moderate)	71/49	135/138	3.155	0.076
Family history (present / absent)	37/83	80/193	0.093	0.760
Mother's academic qualifications (High / Low)	81/39	171/102	0.857	0.355
Father's academic qualifications (High / Low)	76/44	188/85	1.157	0.282
Snoring during sleep (present / absent)	18/102	60/213	2.552	0.110
obesity (present / absent)	18/102	27/246	2.147	0.143
Response to desmopressin treatment				
no response	36	82	-16.226	0.000
partial response	57	62		
complete response	27	129		

Ultimately, 156 (39.69%) children with MNE showed complete response to desmopressin treatment, 119 (30.28%) showed partial improvement, and 118 (30.03%) were unresponsive (Table-1). No differences in age, sex, family history, parent educational level, sleep quality and weight problem were observed between the successful treatment and failed treatment group (Table-2). In the successful treatment group, 63 (40.38%) of children showed a severe MNE phenotype, but in the failed treatment group the percentage of severe symptoms was greater (60.76%, n=144) and this difference was significant (p=0.000) (Table-2). The number of patients who had their first-time visit to the urology clinic in the winter season was 27 (17.31%) in the successful treatment group, and 93 (39.24%) in the failed treatment group. This difference was statistically significant (p=0.000) (Table-2).

**Table 2:** Univariate logistic regression analysis was performed to assess risk factors for desmopressin treatment failure. (n = 393).

Variables		Treatment success group n = 156	Treatment failure group n = 237	p value	OR	95% CI
Age		7.23±1.38	7.51±1.56	0.068	1.138	0.990-1.308
**Gender (male)**						
	Male	85	109	0.100	0.711	0.474-1.067
	Female	71	128			
Mother's age		34.63±5.80	34.84±5.62	0.713	1.007	0.971- 1.043
Father's age		38.36 ±6.25	38.15±5.76	0.726	0.994	0.961- 1.028
**Severity of symptoms**						
	Severe	63	144	0.000	2.286	1.513-3.453
	Mild and moderate	93	93			
**Family history**						
	Present	45	72	0.745	1.076	0.691- 1.677
	Absent	111	165			
**Mother's academic qualifications**						
High		108	156	0.286	0.793	0.519- 1.214
Low		48	81			
**Father's academic qualifications**						
	High	100	133	0.482	0.856	0.555- 1.320
	Low	44	65			
**Snoring during sleep**					
	Present	34	44	0.433	0.818	0.495- 1.351
	Absent	122	193			
**Obesity**						
	Present	17	28	0.780	1.095	0.578- 2.077
	Absent	139	209			
**Visiting clinic**						
	Winter season	27	93	0.000	3.086	1.890-5.036
	Other seasons	129	144			

OR = Odds ratio; CI = confidence interval

Univariate logistic regression analysis showed that both the severity of symptoms and wintertime first-time visit were closely related to desmopressin treatment failure (odds ratio [OR]=2.286, 95% confidence interval [CI]=1.513-3.453, p=0.000 and OR=3.086, 95% CI=1.890-5.036, p=0.000 respectively). Other demographical and clinical factors including age, sex, family history, parent educational level, sleep quality, and overweight did not influence treatment efficacy (Table-2). Similarly, multivariate logistic regression analysis with a forward stepwise model also indicated that the severity of symptoms and wintertime first-time visit were significantly associated with desmopressin treatment failure (OR=2.172, 95% CI=1.423-3.315, p=0.000 and OR=2.945, 95% CI=1.791-4.842, p=0.000 respectively).

## DISCUSSION

Our study confirmed that the complete responsiveness rate and partial responsiveness rate were 39.7% and 30.3% respectively. These data were similar to those obtained in a previous study (14).

Many studies have suggested that seasonal variations in climate are important factors that cannot be ignored during the onset, development, and progression of diseases. Specifically, in regard to enuresis, it has been shown that the duration and frequency of enuresis in the same group increased in the winter compared to that in the summer season (15). Furthermore, in a study by Yoshiyuki Shiroyanagi et al., children with NE received enuresis alarm treatment were more prone to treatment failure in the winter than in any other season, thus showing wintertime as an independent risk factor for treatment failure (9). These findings were similar to those of our current study. In fact, we showed that treatment efficacy in children who had their first-time visit and began desmopressin treatment in the winter had poorer outcomes than children that did so during other seasons. The possible explanations for this pattern could be the following: first, cold temperatures can increase the osmolality of urine, which could be attributed to a reduced arginine vasopressin (AVP) excretion from the hypophysis as well as to the failure to maintain the concentration gradient in the renal medulla (16, 17). Also, systemic levels of cyclic guanosine monophosphate (cGMP) are significantly blunted in the cold, indicating suppressed nitric oxide (NO) signaling (17). The central nervous system has been confirmed to be capable of secreting anti-diuretic hormone (ADH) in response to NO stimulation (18); thus, decreased NO secretion during the cold months could lead to a reduction in ADH production. However, another study demonstrated that cold-induced diuresis was not a consequence of a reduction in AVP release, because no change in AVP plasma levels was recorded in rats. Rather, cold-induced diuresis was possibly caused by the downregulation of V2 receptors and the subsequent decrease in the expression of the AQP-2 water channel protein (19). The limitation of this study was that data were obtained in rats; consequently, whether the same mechanism occurs in the human body has yet to be determined.

The second possible explanation for the high rate of desmopressin treatment failure in the winter season is the negative influence of cold weather on bladder capacity. Increased nocturnal detrusor activity and low bladder capacity play important roles in nocturnal enuresis (11). Studies have demonstrated that cold stressors increased detrusor activity and decreased bladder capacity without altering body temperature in rats (20, 21). A human study showed that lower urinary tract symptoms were significantly improved by physical exercise in cold sensitive patients (22). In this report, although desmopressin reduced urine production, the increased nocturnal detrusor activity and low bladder capacity induced by the cold remained unresolved. Therefore, the findings of the abovementioned study could explain why a higher rate of desmopressin treatment failure is observed during the winter season.

Of course, the present study had several limitations. First, the sample size was small. Second, the retrospective design of the study imposed inherent limitations. Our findings need to be confirmed by a large-scale, prospective study.

In conclusion, winter season and severe symptom of enuresis were associated with desmopressin treatment failure in MNE.
